# Targeting Fatty Acid Reprogramming Suppresses CARM1-expressing Ovarian Cancer

**DOI:** 10.1158/2767-9764.CRC-23-0030

**Published:** 2023-06-20

**Authors:** Simona Lombardi, Aaron R. Goldman, Hsin-Yao Tang, Andrew V. Kossenkov, Heng Liu, Wei Zhou, Meenhard Herlyn, Jianhuang Lin, Rugang Zhang

**Affiliations:** 1 Immunology, Microenvironment and Metastasis Program, The Wistar Institute, Philadelphia, Pennsylvania.; 2 Department of Pharmacy and Biotechnology, University of Bologna, Bologna, Italy.; 3 Molecular and Cellular Oncology Program, The Wistar Institute, Philadelphia, Pennsylvania.; 4 Gene Expression and Regulation Program, The Wistar Institute, Philadelphia, Pennsylvania.; 5 Department of Experimental Therapeutics, University of Texas MD Anderson Cancer Center, Houston, Texas.

## Abstract

The arginine methyltransferase CARM1 exhibits high expression levels in several human cancers, with the trend also observed in ovarian cancer. However, therapeutic approaches targeting tumors that overexpress CARM1 have not been explored. Cancer cells exploit metabolic reprogramming such as fatty acids for their survival. Here we report that CARM1 promotes monounsaturated fatty acid synthesis and fatty acid reprogramming represents a metabolic vulnerability for CARM1-expressing ovarian cancer. CARM1 promotes the expression of genes encoding rate-limiting enzymes of *de novo* fatty acid metabolism such as acetyl-CoA carboxylase 1 (ACC1) and fatty acid synthase (FASN). In addition, CARM1 upregulates stearoyl-CoA desaturase 1 (SCD1) that produces monounsaturated fatty acid by desaturation. Thus, CARM1 enhances *de novo* fatty acids synthesis which was subsequently utilized for synthesis of monounsaturated fatty acids. Consequently, inhibition of SCD1 suppresses the growth of ovarian cancer cells in a CARM1 status–dependent manner, which was rescued by the addition of monounsaturated fatty acids. Consistently, CARM1-expressing cells were more tolerant to the addition of saturated fatty acids. Indeed, SCD1 inhibition demonstrated efficacy against ovarian cancer in both orthotopic xenograft and syngeneic mouse models in a CARM1-dependent manner. In summary, our data show that CARM1 reprograms fatty acid metabolism and targeting SCD1 through pharmacological inhibition can serve as a potent therapeutic approach for CARM1-expressing ovarian cancers.

**Significance:**

CARM1 reprograms fatty acid metabolism transcriptionally to support ovarian cancer growth by producing monounsaturated fatty acids, supporting SCD1 inhibition as a rational strategy for treating CARM1-expressing ovarian cancer.

## Introduction

Epithelial ovarian cancer (EOC) is most lethal among gynecologic malignancies in the United States (1). High-grade serous ovarian cancer (HGSOC) is the most prevalent subtype of EOC accounting for >70% of cases and a substantial amount of the mortalities caused by EOC. HGSOC is genetically characterized by different subgroups. Therefore, it is important to develop therapeutic strategies tailored for distinct molecular subsets of HGSOC (2). Analysis of The Cancer Genome Atlas (TCGA) reported that within spontaneous HGSOC *CARM1* is amplified in approximately 10% of cases and overexpressed in an additional approximately 10% of cases (3). CARM1 is a type I protein arginine methyltransferase that uses its catalytic function to asymmetrically dimethylate arginine residues on histone and non-histone protein substrates (4). In addition to its catalytic activity, CARM1 functions as a transcription coactivator for expression of genes including those driven by XBP1s in response to endoplasmic reticulum (ER) stress in an enzymatic activity–independently manner (5). Elevated expression levels of CARM1 have been observed in multiple cancer types such as breast, colon, and prostate suggesting a potential oncogenic role for CARM1 in the context of human cancers (6–8). Notably, *CARM1* is most amplified in ovarian cancer based on TCGA database. However, the potential that CARM1 might constitute a unique therapeutic vulnerability remains to be fully explored.

Fatty acids (FA) are essential for cell survival because they are key structural constituents of cell membranes and important signaling components by functioning as the critical components for various lipid species (9). FA is comprised of a carboxylic acid group and a hydrocarbon chain with varying carbon chain lengths and desaturation degrees. In normal tissues, *de novo* lipogenesis is typically confined in adipocyte and hepatocytes. However, tumor cells can reactivate this pathway to support their proliferation. Cytoplasmatic acetyl-CoA is the principal substrate for FAs synthesis to be converted into malonyl-CoA through irreversible carboxylation by acetyl-CoA carboxylases (ACC). Next, the fatty acid synthase (FASN) catalyze the condensation of one molecule of acetyl-CoA and seven malonyl-CoA molecules to generate the saturated 16-carbon FA palmitate. Palmitate can be further desaturated by stearoyl-CoA desaturase 1 (SCD1) to produce monounsaturated FAs (MUFA). Palmitate is the primary end-product of *de novo* lipogenesis and is used to generate additional FA species such as sterate and oleate. These FAs subsequently are used to generate more complex lipids. SCD1 activity is critical for cancer cells. Recent evidence supports that SCD1 inhibition impairs cell proliferation by perturbing the balance between unsaturated and saturated FAs, while accumulation of saturated FAs mediates cell death induced by SCD1 inhibition (10). Notably, FAs are altered in ovarian cancer (11). However, the mechanism underlying FAs reprogramming in ovarian cancer remains poorly understood. In the current study we show that CARM1 promotes *de novo* FAs and subsequent MUFA synthesis, and SCD1 inhibition represents a therapeutic strategy for CARM1-expressing ovarian cancer.

## Materials and Methods

### Cell Culture, Transfection, and Reagents

UPK10 (gift from Dr. J. Conejo-Garcia, Moffitt Cancer Center, Tampa, FL) and HEK293FT cells (RRID:CVCL_6911) were cultured in DMEM supplemented with 10% FBS and penicillin/streptomycin (1%) with 5% CO_2_ at 37°C. Human ovarian cancer cell lines A1847 (RRID:CVCL_9724), PEO4 (RRID:CVCL_2690), CAOV3 (RRID:CVCL_0201), OVCAR3 (RRID:CVCL_DH37), OVCAR10 (RRID:CVCL_4377), and PEO1 (RRID:CVCL_2686) were obtained from ATCC and were cultured in RPMI1640 supplemented with 10% FBS and penicillin/streptomycin (1%) with 5% CO_2_ at 37°C. Cell lines were recovered from liquid nitrogen and cultured for at least two passages before used for experiment. Short tandem repeat (STR) analysis was performed by the The Wistar Institute Genomics Facility for the authentication of the cell line based on the available STR profiles. Monthly, *Mycoplasma* testing was conducted using LookOut *Mycoplasma* PCR detection (Sigma) until 1 week before the end of the experiments. Lipofectamine 2000 (Life Technologies) was used to transfect the cells according to manufacturer's manual. SCD1 inhibitor CAY10566 (#HY-15823) and CARM1 inhibitor EZM2302 (#HY-111109) were purchased from MCE. BSA-palmitate was purchased from Cayman (#29558) and BSA-oleic acid from Sigma (#O3008).

### CRISPR-mediated Knockouts

pLentiCRISPR v2 (RRID: Addgene_52961) was used to generate the construct to knock out CARM1 as published previously (5). Briefly, pLentiCRISPR v2 (RRID: Addgene_52961) was digested with restriction enzyme *BsmBI* (NEB), then separated in the agarose gel (1%). The correct DNA band in agarose gel was cut and purified by QIAquick gel extraction kit (QIAGEN, catalog no.166047244). gDNA oligo pairs were phosphorylated by T4 PNK M0201S; NEB) in T4 ligation buffer (NEB) and annealed in a thermocycler with slow temperature ramp down from 95°C to room temperature at 5°C/minute. The ligation between the digested pLentiCRISPR v2 plasmid and the annealed gDNA (diluted 1:200 in RNase/Dnase-free water) was performed using Quick Ligase (NEB). Human *CARM1* gRNA (5′-AGCACGGAAAATCTACGCGG-3′) and mouse *Carm1* gRNA (5′-TCGCGTCGCCGATAGTGAGG-3′) were used for cloning.

### Lentivirus Infection

HEK293FT cell line were transfected with pLentiCRISPR-v2-gCARM1, psPAX2 (RRID: Addgene_12260), and pCMV-VSV (RRID: Addgene_8454) by Lipofectamine 2000 for 6 hours and replaced with fresh medium. A total of 72 hours after transfection, virus containing supernatant medium was collected, filtered (0.45 μm filter), and used for cells infection. Puromycin (1 μg/mL) was added for selection at 48 hours after infection.

### Immunoblots

Immunoblots were performed as described previously (5). Briefly, cell pellets was suspended in RIPA lysis buffer (5). Protein concentration was determined using bicinchoninic acid assay (Pierce). Equal amount of total protein was loaded and separated by SDS-PAGE and transferred onto polyvinylidene fluoride membrane (Millipore). The membranes were blocked with 4% BSA/Tris-buffered saline with 0.1% Tween 20 detergent (TBS-T) and then probed with corresponding antibodies. The following antibodies were used: mouse anti-β-actin (Sigma, #A5316, RRID:AB_476743), rabbit anti-SCD1 (Abcam, #ab236868, RRID: AB_2928123), rabbit anti-CARM1 (Cell Signaling Technology, #3379S, RRID:AB_2068433), rabbit anti-FASN (Cell Signaling Technology, #3189, RRID:AB_2100798), rabbit anti-ACC1 (Cell Signaling Technology, #4190, RRID:AB_10547752), rabbit anti-cleaved PARP (Cell Signaling Technology, #5625S, RRID:AB_10699459), and rabbit anti-Lamin A/C (Cell Signaling Technology, #2032S, RRID:AB_2136278).

### qRT-PCR

Total RNA was purified with RNeasy Kit (Qiagen #74004) according to the manufacturer's manual. RT-PCR was performed using the High-Capacity cDNA Reverse Transcription Kit (Thermo Fisher Scientific). Real-time quantitative PCR (qPCR) reaction was prepared with using the iTaq Universal SYBR Green Supermix (Bio-Rad) and run on the Real-Time PCR System QuantStudio 3. PCR primer sequences are: *FASN* forward: 5′-CCTGGCTGCCTACTACATCG-3′ and reverse: 5′-CACATTTCAAAGGCCACGCA-3′; *ACC1* forward: 5′-CATCTCCCTTGGCCCAACC-3′ and reverse: 5′-TCTGAGCCAACAGAAGCAGG-3′; *SCD1* forward: 5′-CTTGCGATATGCTGTGGTGC-3′ and reverse: 5′-CCGGGGGCTAATGTTCTTGT-3′; *GAPDH* forward: 5′-GTCTCCTCTGACTTCAACAGCG-3′ and reverse: 5′-ACCACCCTGTTGCTGTAGCCAA-3′.

### Colony Formation Assay

Initially, 3,000 cells were seeded into each well of a 24-well tissue culture plate, then treated with the specified compound and concentration. The cell medium was refreshed every 3 days maintaining the specified compound doses for a period of 10 days. After 10 days, colonies were stained with 0.05% crystal violet/10% methanol and quantified using the NIH ImageJ software (RRID: SCR_003070).

### Chromatin Immunoprecipitation and Chromatin Immunoprecipitation Sequencing

Chromatin immunoprecipitation (ChIP) was performed as described previously (5). Briefly, the cells were fixed with 1% formaldehyde/PBS (10 minutes at room temperature) followed by quenching with 0.125 mol/L glycine. The fixed cells were lysed in ChIP lysis buffer 1 (5) on ice for 30 minutes and lysis buffer 2 (5) at room temperature for 10 minutes. Chromatin was fragmented using MNase (Cell Signaling Temperature) for 15 minutes at 37°C. The cells were broken down by a single pulse of bioruptor, high output for 30 seconds. Following centrifugation, the digested chromatin from each sample was collected and subjected to overnight incubation at 4 °C with the following antibodies: CARM1 (Cell Signaling Technology #12495S, RRID: AB_2797935); H3R17me2a (Abcam #ab8284, RRID: AB_306434); and XBP1s (Novus Biological #NBP1-77681, RRID: AB_11010815). Then next day, the antibody and target protein-DNA complex was captured by Protein A/G Dynabeads for 1.5 hours incubation at 4°C. After incubation, the Dynabeads were washed and the chromatin was eluted with TES buffer [1 mmol/L ethylenediaminetetraacetic acid (EDTA), 1% SDS, 10 mmol/L Tris-Cl (pH 8.0)]. The eluted DNA/protein complexes were subject to proteinase K digestion and de-cross-link. The remaining target DNA was purified with the Zymo ChIP DNA Clean and Concentrator Kit (Zymo Research, catalog no. D5205). The ChIP-qPCR primers sequences are as follows: *FASN* forward: 5′-CGGGGAAAGCCACCAACA-3′ and reverse: 5′-GCTCCTCCAGGCCCTTCA-3′; *ACC1* forward: 5′-GTGAACGGCCTGGAGTAACC-3′ and reverse: 5′-CCCCTGTCTCCCACCTCAG-3′; *SCD1* forward: 5′-AGAGGGAACAGCAGATTGCG-3′ and reverse: 5′-CTGTAAACTCCGGCTCGTCA-3′.

For chromatin immunoprecipitation sequencing (ChIP-seq), the NEBNext Ultra DNA Library Prep Kit (NEB; #E7645) was used for library construction using purified DNA above according to the manufacturer's instructions. The Wistar Genomic facility conducted a 75-bp single-end run on the Next Seq 500 (Illumina) for sequencing the libraries.

### Intrabursal Orthotopic Xenograft Model *In Vivo*

*In vivo* protocol for animal experiments were reviewed and approved by the Institutional Animal Care and Use Committee, of The Wistar Institute. Female C57BL/6 mice and NSG were purchased from Charles River Laboratories and The Wistar Institute Animal Facility, respectively. 0.5 × 10^6^ wildtype or CARM1 knockout UPK10 or A1847 cells were injected unilaterally into the *bursa sac* of the ovary of 6–8 weeks old C57BL/6 or NSG mice (*n* = 5 mice/group, except *n* = 3 per group for CARM1 knockout UPK10 xenograft in C57BL/6 mice; refs. 5, 12). After tumors were formed, mice were randomized. Randomized groups were treated with SCD1 inhibitor CAY10566 (MedChemExpress, #HY-15823; 5 mg per kg in 0.5% methyl cellulose, once per day by oral gavage) or vehicle for 2 weeks. Following treatment, the tumors were surgically excised, and the tumor weight was assessed as indicator for tumor burden.

### IHC Staining

Serial dissection of tumors, fixed in PBS (10% formalin; Thermo Fisher Scientific, #SF100-4), and embedded in paraffin were subject to IHC staining performed using Dako EnVision^+^system as described previously (5). Briefly, the sections were deparaffinized and rehydrated, then the endogenous peroxidase activity was quenched by 3% hydrogen peroxide in methanol. The sections were subjected to antigen retrieval performed in sodium citrate buffer (Thermo Fisher Scientific, #005000). Next, each section was blocked with PBS/1% BSA. The following primary antibodies were used for overnight incubation at 4°C: cleaved caspase 3 (Cell Signaling Technology, #9661, RRID: AB_2341188, 1:200), Ki67 (Cell Signaling Technology, #9449, RRID: AB_2797703, 1:1,000), CARM1 (Cell Signaling Technology, #12495, RRID: AB_2797935, 1:100), SCD1 (Proteintech, #23393-1-AP, RRID: AB_2744674, 1:100), FASN (Cell Signaling Technology, #3189, RRID: AB_2100798), or ACC1 (Cell Signaling Technology, #4190, RRID: AB_10547752). Mayer's Hematoxylin (Dako, #3309S) was used for counterstaining. Histologic score (H score) was used to assess the target expression.

### Reverse Phase Protein Array

A total of 0.5 × 10^6^/3 mL wildtype or CARM1 knockout A1847 or PEO4 cells were seeded onto 6-well plates for 24 hours. The cells were washed with PBS and lysed in 100 μL lysis buffer (50 mmol/L HEPES pH 7.4, 1% Triton X‐100, 1.5 mmol/L MgCl_2_, 150 mmol/L NaCl, 100 mmol/L NaF, 1 mmol/L ethylene glycol-bis(β-aminoethyl ether)-N,N,N′,N′-tetraacetic acid (EGTA), 10 mmol/L sodium pyrophosphate, 10% glycerol, and 1 mmol/L Na_3_VO_4_), with added phosphatase and protease inhibitors (Roche Applied Science, catalog nos. 04906837001 and 05056489001, respectively) for 20 minutes on ice with occasional shakings. The cells were then scraped off the wells and the cell lysates were centrifuged (14,000 rpm, at 4°C for 10 minutes). The supernatants were collected, then the protein concentrations were measured by BCA and adjusted to 1.5 μg/μL. Lysates were mixed with 4x SDS sample buffer (8% SDS, 10% beta-mercaptoethanol, 40% Glycerol, and 0.25 mol/L Tris‐HCL pH 6.8) and boiled at 95°C for 5 minutes. Samples were submitted to reverse phase protein array (RPPA) core facility (MD Anderson Cancer Center).

### Metabolomics Analysis

Metabolomics analysis was carried out following a previously described method (13). Summarizing, polar metabolites were extracted using 80% methanol. The next LC/MS analysis was performed using the mass spectrometer Thermo Fisher Scientific Q Exactive HF-X set with HESI II probe. The device was coupled to a Thermo Fisher Scientific Vanquish Horizon Ultra-High-Performance Liquid Chromatography (UHPLC) system. Chromatographic separation was performed by hydrophilic interaction liquid chromatography (HILIC) at 0.2 mL/minute at 45°C with ZIC-pHILIC column (2.1-mm i.d. × 150-mm; EMD Millipore) using solven A (0.1% ammonium hydroxide pH 9.2, 20 mmol/L ammonium carbonate), and solvent B (acetonitrile) with a gradient of: 0 minute-85% B; 2 minutes-85% B; 17 minutes-20% B; 17.1 minutes-85% B; and 26 minutes-85% B. Next, the samples were randomized and analyzed for quantification using full mass spectrometry (MS) scans with polarity switching using a scan range of 65 to 975 m/z, automated gain control (AGC) target of 1E6, 120,000 resolution, and a maximum injection time of 100 ms. Next, combining equal volume of each samples a sample pool (QC) was created and then periodically analyzed throughout the run sequence with full MS scans. MS-MS analysis was also performed for the QC samples (using different runs for negative and positive mode analysis) as follows: full MS scan was obtained as described above and followed by MS-MS of the 10 most abundant ions (AGC target of 5E4, 15,000 resolution, an isolation width of 1.0 m/z, max IT of 50 ms, and a stepped collision energy of 20, 40, and 60). The identification of the metabolites (MS-MS data) and quantitation (MS only data) were obtained using Compound Discoverer 3.0 (Thermo Fisher Scientific). The metabolites were therefore identified by accurate mass and either retention time using a mass list generated from standards or by MS-MS spectra using the mzCloud database (accessible at www.mzcloud.org), with putative annotations with a matched score of 50 at least. Metabolits levels were normalized to the protein amount for each sample.

### Global Lipidomics and FA Saturation Analysis

Control or CARM1 knockout A1847 cells were washed with cold PBS, scraped into cold methanol, and spiked with EquiSPLASH mix (Avanti Polar Lipids). Lipids were extracted using a modified Folch extraction (2:1:1 chloroform:methanol:0.88% sodium chloride) and analyzed by LC/MS-MS. LipidSearch 4.2 software (Thermo Fisher Scientific) detected lipid species based on MS-MS spectra with product ion mass tolerances and 5 ppm precursor. The identification of lipid species was filtered on the basis of the expected identification quality and main adduct. The peak areas were utilized for quantification and were adjusted by EquiSPLASH lipids to represent the respective classes. In addition, the values were normalized to the protein content in each sample. The quantification of lipid classes was performed by summing peak areas of all species in the same class. Regarding the saturation analysis, the FAs incorporated into lipids with high-confidence identifications (grades A and B from LipidSearch) were classified in accordance with the number of carbon double-bonds as saturated (0), monounsaturated (1), or polyunsaturated (>1). The quantification of each lipid species with FA level identification was weighted by the number of FAs of each type present in the specie.

### Total FA Quantification

Lipids were saponified with methanolic KOH and FAs were extracted using hexane. Total FA extracts were analyzed by the mass spectrometer LC/MS on a Thermo Fisher Scientific Q-Exactive HF-X and a Vanquish Horizon UHPLC system. Reversed-phase chromatography utilized the Accucore C18 column with size 2.1 mm × 150 mm (Thermo Fisher Scientific), using water and acetonitrile solvents (both including 0.1% acetic acid). Next, full MS scans were acquired in negative mode (from 180 to 650 m/z at 120,000 resolution). The FAs were identified using TraceFinder 4.1 (Thermo Fisher Scientific) by accurate retention time and mass that derived from the standards and quantified peak area. FA levels of each sample were corrected by the level of deuterated FA 18:d7 that is derived from saponified EquiSPLASH lipids and further normalized on the basis of the protein amount.

### Kyoto Encyclopedia of Genes and Genomes Pathway Analysis

The RNA sequencing (RNA-seq) data for wildtype and CARM1 knockout A1847 cells were downloaded from previously published NCBI Gene Expression Omnibus (GEO) dataset (GSE95644; ref. 12). Differentially expressed genes (fold change >2; FDR < 0.05) were subjected to Kyoto Encyclopedia of Genes and Genomes (KEGG) pathway analysis using DAVID platform (https://david.ncifcrf.gov/tools.jsp).

### Statistical and Bioinformatics Analysis

*Bowtie* was used to aligned ChIP-seq data (14) against human genome version hg19. *HOMER* (15) was utilized for generating bigwig files using default normalization parameters.

The results are presented as mean ± SEM resulting from three independent experiments. Unpaired, two-tailed Student *t* test was utilized to performed two-group comparative analyses. GraphPad software package (Prism 9.0, RRID: SCR_002798) for Windows were used to performed all the statistical analyses. *P* < 0.05 was considered statistically significant.

### Data Availability

The ChIP-seq data generated in the study have been deposited in the GEO (RRID: SCR_005012) under accession no. GSE202259. XBP1s CUT&RUN sequencing was downloaded from GEO database under accession GSE157118. TCGA high-grade serous ovarian cancer RNA-seq dataset was downloaded from cBioPortal.

## Results

### CARM1 Regulates FA Metabolic Pathway

**FIGURE 1:**
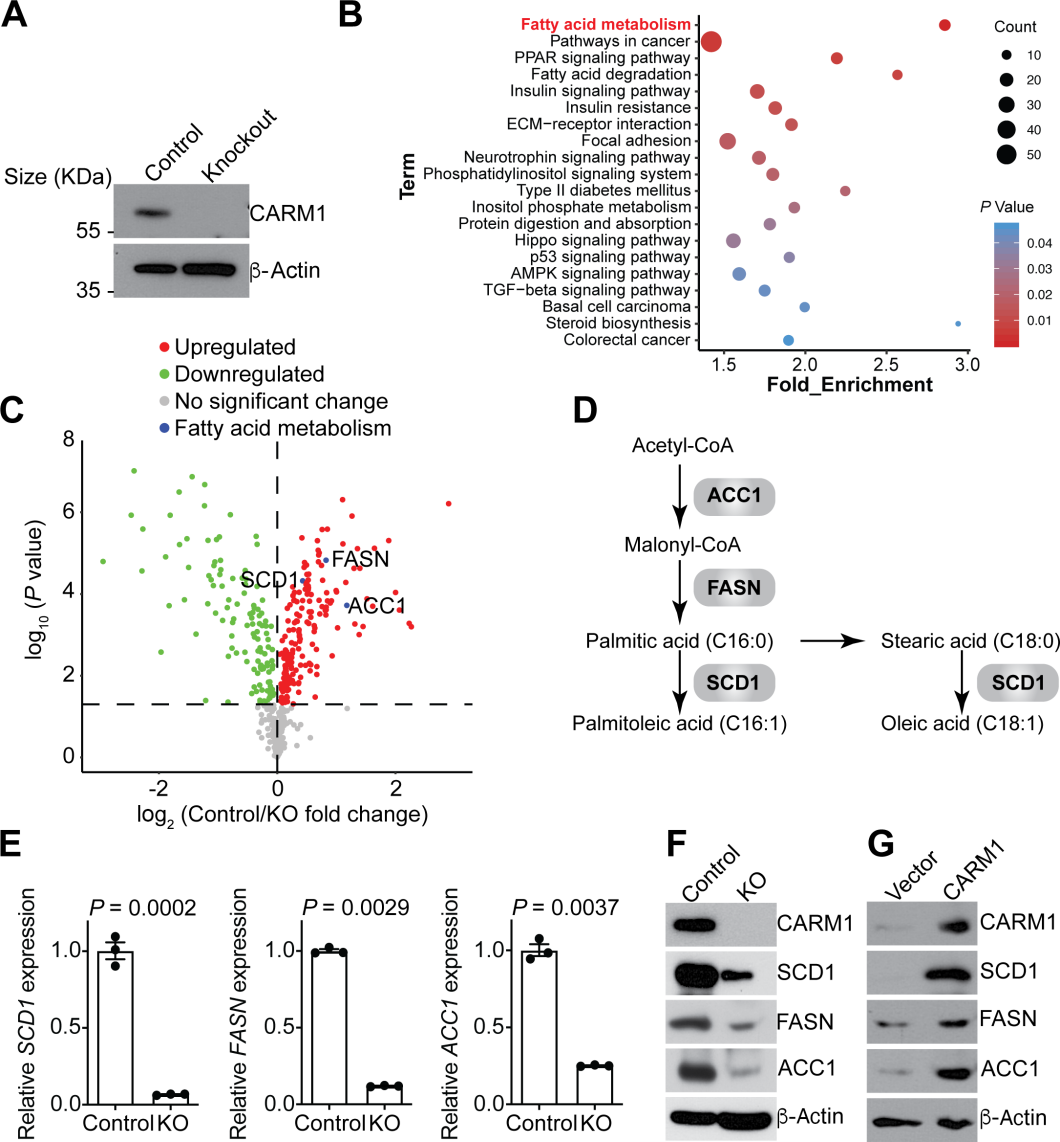
CARM1 regulates genes involved in FA metabolism. **A,** Expression of CARM1 and a loading control β-actin in control and CARM1 knockout (KO) A1847 cells determined by immunoblot. **B,** Scatter plot of KEGG pathway analysis for genes upregulated in wildtype compared with CARM1 KO A1847 cells (WT/KO >2, FDR < 0.05). Dot size represents gene number, color represents *P* value. *P* values were calculated by KEGG analysis. **C,** Volcano plot illustrating the protein expression detected by RPPA in wildtype or CARM1 KO A1847 cell lysates. Red dots and green dots indicate protein expression significantly upregulated in wildtype and knockout cells, respectively. SCD1, FASN, and ACC1 were highlighted in blue. *P* value was calculated using a two-tailed Student *t* test. **D,** Schematic of the MUFA synthesis pathway. **E,** mRNA expression of *ACC1*, *FASN,* and *SCD1* in control and CARM1 KO A1847 cells determined by qRT-PCR analysis. *P* value was calculated using a two-tailed Student *t* test. **F,** Expression of SCD1, FASN, ACC1 and a loading control β-actin in control and CARM1 KO A1847 cells as determined by immunoblot. **G,** Expression of SCD1, FASN, ACC1 and a loading control β-actin in control and CARM1 overexpressed CAOV3 cells as determined by immunoblot. *P* value was calculated using a two-tailed Student *t* test. Data represent mean ± SEM, *n* = 3 biologically independent experiments.

To systematically explore the pathways that are regulated by CARM1, we performed KEGG pathway analysis using differentially expressed genes in CARM1-expressing and the isogenic CARM1 knockout A1847 cells based on RNA-seq datasets (Fig. 1A and B). On the basis of the analysis, the FA metabolism is the top pathway enriched among the gene regulated by CARM1 (Fig. 1B; Supplementary Fig. S1A). In addition, we conducted a profile analysis of the protein expression changes induced by CARM1 knockout in A1847 cell line using RPPA. Consistently, the key regulatory enzymes of the FA metabolism pathway such as SCD1, FASN, and ACC1 were significantly downregulated by CARM1 knockout (Fig. 1C and D; Supplementary Table S1). The similar downregulation of SCD1, FASN, and ACC1 by CARM1 knockout was detected in another CARM1 high–expressing cell line PEO4 (Supplementary Fig. S1B; Supplementary Table S2). This suggests that the observed downregulation of SCD1, FASN and ACC1 is not a cell line specific effect. We validated that downregulation of SCD1, FASN, and ACC1 at both mRNA and protein levels by CARM1 knockout in A1847 and PEO4 cell lines (Fig. 1E and F; Supplementary Fig. S1C and S1D). Conversely, overexpression of CARM1 in CARM1 low–expressing CAOV3 cells upregulated SCD1, FASN, and ACC1 (Fig. 1G). Consistently, there was a positive correlation between *CARM1* expression and expression of *SCD1*, *FASN,* and *ACC1* in the HGSOC TCGA dataset (Supplementary Fig. S1E). Thus, CARM1 regulates FA metabolic pathway by promoting the expression of key enzymes SCD1, ACC1, and FASN.

**FIGURE 2:**
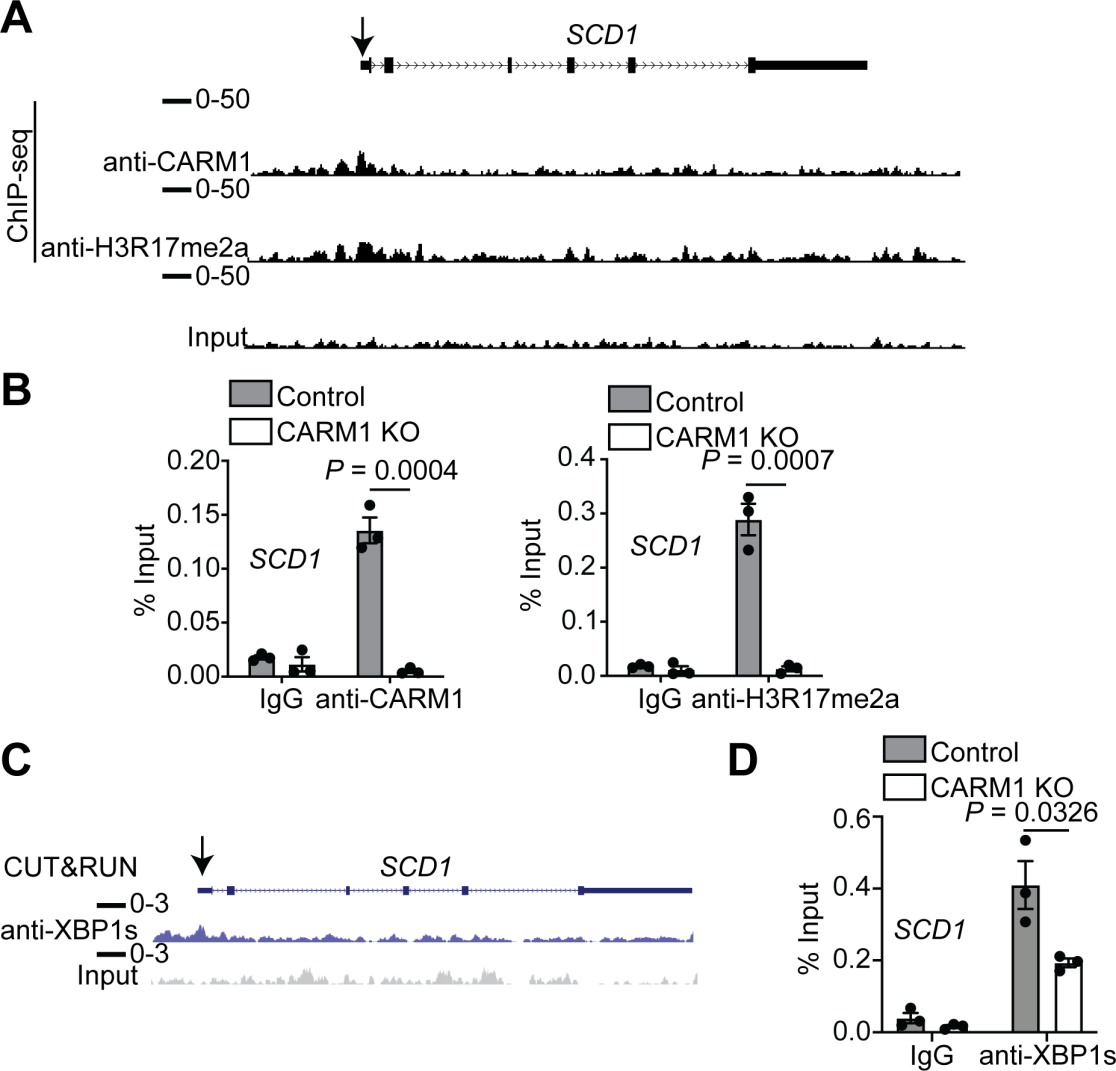
CARM1 enhances SCD1 expression by recruiting XBP1s to their promoters independently of its enzymatic activity. **A,** ChIP-seq track of CARM1 and H3R17me2a in the *SCD1* promoter. The arrow indicates the CARM1 and H3R17me2a peak in the promoter region of *SCD1* gene. **B,** ChIP-qPCR analysis for the binding of CARM1 and H3r17me2a or negative control IgG at the *SCD1* promoter in control and CARM1 KO A1847 cells. **C,** CUT&RUN track of XBP1s in the *SCD1* promoter. The arrow indicates the XBP1s peak in the promoter region of *SCD1* gene. **D,** ChIP-qPCR analysis for the binding of XBP1s or negative control IgG at the *SCD1* promoter in control and CARM1 KO A1847 cells. *P* value was calculated using a two-tailed Student *t* test. Data represent mean ± SEM, *n* = 3 biologically independent experiments.

We next aimed to investigate the underlying mechanism through which CARM1 modulates the expression of SCD1, FASN, and ACC1. Toward this goal, we performed ChIP-seq analysis for both CARM1 and its enzymatic product H3R17me2a that is known to regulate gene transcription (4). Indeed, the ChIP-seq analysis revealed that CARM1 and H3R17me2a were associated with *SCD1*, *FASN,* and *ACC1* promoters (Fig. 2A; Supplementary Fig. S2A). Accordingly, we validated the association of H3R17me2a and CARM1 in CARM1-expressing cells (Fig. 2B; Supplementary Fig. S2B and S2C). Notably, the association of both H3R17me2a and CARM1 with their promoters was reduced by CARM1 knockdown (Fig. 2B; Supplementary Fig. S2B and S2C). This suggests that CARM1 may regulate the expression of SCD1, FASN, and ACC1 through its enzymatic activity. To examine this hypothesis, CARM1-expressing A1847 cell lines were treated with EZM2302, a highly specific inhibitor of CARM1 methyltransferase activity. Indeed, EZM2302 reduced the levels of the product of CARM1 activity H3R17me2a (Supplementary Fig. S2D). In contrast, EZM2302 failed to downregulate SCD1, FASN, and ACC1 (Supplementary Fig. S2D and S2E), supporting the notion that CARM1 regulates their expression independently of its enzymatic activity.

Notably, CARM1 can function as a transcription coactivator by recruiting XBP1s in an enzymatic activity–independent manner during ER stress response (5). In addition, previous studies established that XBP1s is crucial for the regulating of FA metabolism by promoting the transcription of genes such as *SCD1* (16). These findings raised that possibility that CARM1 could act as a coactivator of XBP1s to upregulate SCD1, FASN, and ACC1. Indeed, XBP1s is associated with the promoters of *SCD1*, *FASN*, and *ACC1* based on a published CUT&RUN analysis in A1847 cell line (Fig. 2C; Supplementary Fig. S2F; ref. 5). We next validated the association of XBP1s with the promoters of *SCD1*, *FASN*, and *ACC1* by ChIP analysis (Fig. 2D; Supplementary Fig. S2G). XBP1s association with the promoter of these genes is decreased by CARM1 knockout (Fig. 2D; Supplementary Fig. S2G), supporting a model whereby CARM1 enhances the expression of these genes by recruiting XBP1s to their promoters independently of its enzymatic activity. These findings are consistent with the literature that both CARM1 and FA metabolism play a key role in the ER stress response (5, 16).

### CARM1 Reprograms FA Metabolism

**FIGURE 3:**
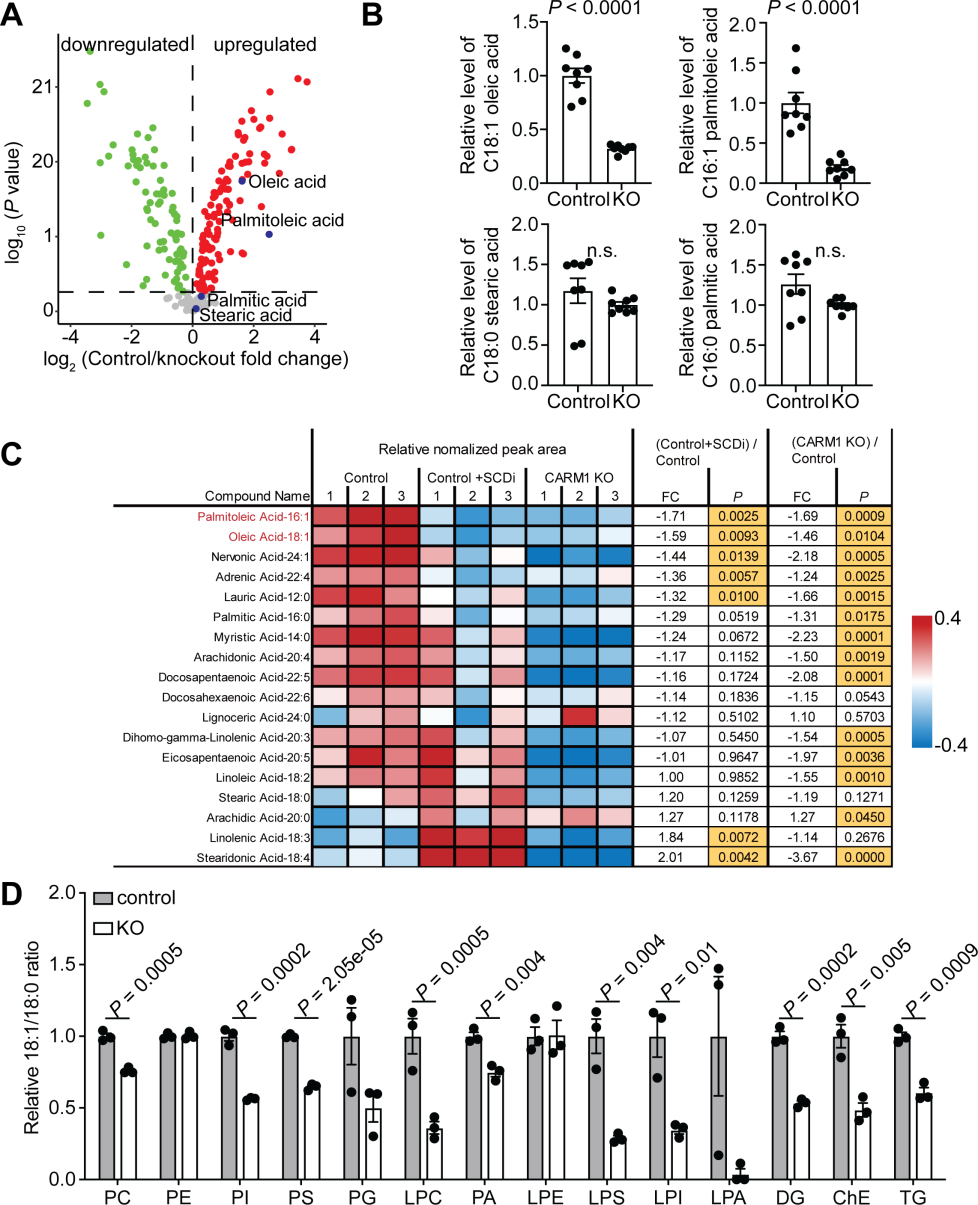
CARM1 promotes MUFA synthesis. **A,** Volcano plot illustrating the changes of metabolite levels in wildtype or CARM1 KO A1847 cells detected by LC/MS. Red dots and green dots indicate metabolites significantly upregulated or downregulated in wildtype compared with knockout cells, respectively. Free oleic acid, palmitoleic acid, palmitic acid, and stearic acid were highlighted in blue. *P* value was calculated using a two-tailed Student *t* test. **B,** The level of free oleic acid (18:1), palmitoleic acid (16:1), stearic acid (18:0), and palmitic acid (16:0) as shown in A. **C,** Changes in total FA profiling for control treated vehicle or SCD1 inhibitor 5 μmol/L CAY10566 for 48 hours, or CARM1 KO A1847 cells. **D,** The ratio between total oleic acid (18:1) and stearic acid (18:0) in different lipid species determined by lipid profiling in wildtype and CARM1 KO A1847 cells. PC: Phosphatidylcholine; PE: phosphatidylethanolamine; PI: Phosphatidylinositol; PS: Phosphatidylserine; PG: Phosphatidylglycerol; LPC: Lysophosphatidylcholine; PA: Phosphatidic acid; LPE: lysophosphatidylethanolamine; LPS: Lysophosphastidylserine; LPI: Lysophosphatidylinositol; LPA: Lysophosphatidic acid; DG: Diglyceride; ChE: cholesterol ester; and TG:Triglyceride. *P* value was calculated using a two-tailed Student *t* test. Data represent mean ± SEM, *n* = 3 biologically independent experiments unless otherwise stated.

We next profiled the changes in global metabolites induced by CARM1 using LC/MS-MS analysis. The analysis revealed that free MUFAs such as oleic (18:1) and palmitoleic (16:1) acids were significantly downregulated by CARM1 knockout (Fig. 3A and B). Interestingly, the free saturated FAs such as steric (18:0) and palmitic (16:0) acids were not significantly affected by CARM1 knockout (Fig. 3A and B). This result suggests that CARM1-induced increase in *de novo* FA synthesis through key enzymes such as ACC1 and FASN was further utilized to generate MUFAs by the upregulated SCD1. To test this possibility, we profiled changes in total FAs induced by either CARM1 knockout or SCD1 inhibition by CAY10566 (17). Indeed, both CARM1 knockout and SCD1 inhibition significantly decreased the levels of MUFAs such as palmitoleic (16:1) and oleic (18:1) acids (Fig. 3C). In contrast, only CARM1 knockout significantly decreased the total saturated FAs such as steric acid (18:0) and palmitic acid (16:0) levels (Fig. 3C). Thus, despite the increase in total FA synthesis induced by CARM1 (Fig. 3C), the free saturated FAs were not increased by CARM1 expression due to their utilization by SCD1 to produce MUFAs (Fig. 3A and B).

Given the importance of FAs in lipid biogenesis, we next profiled changes in lipids induced by CARM1 knockout. Consistent with the upregulation of MUFAs by CARM1, their incorporation into lipids was significantly reduced by CARM1 knockout (Fig. 3D; Supplementary Fig. S3A). In addition, SCD1 is known to promote the biosynthesis of cholesterol ester and triglycerides (18, 19). Consistently, the levels of cholesterol ester and triglycerides were significantly regulated by CARM1 knockout (Supplementary Fig. S3B). Collectively, these findings support that CARM1 promotes FA metabolism to increase MUFA synthesis through upregulating SCD1.

### SCD1 Inhibition is Selective Against CARM1 Expression

**FIGURE 4:**
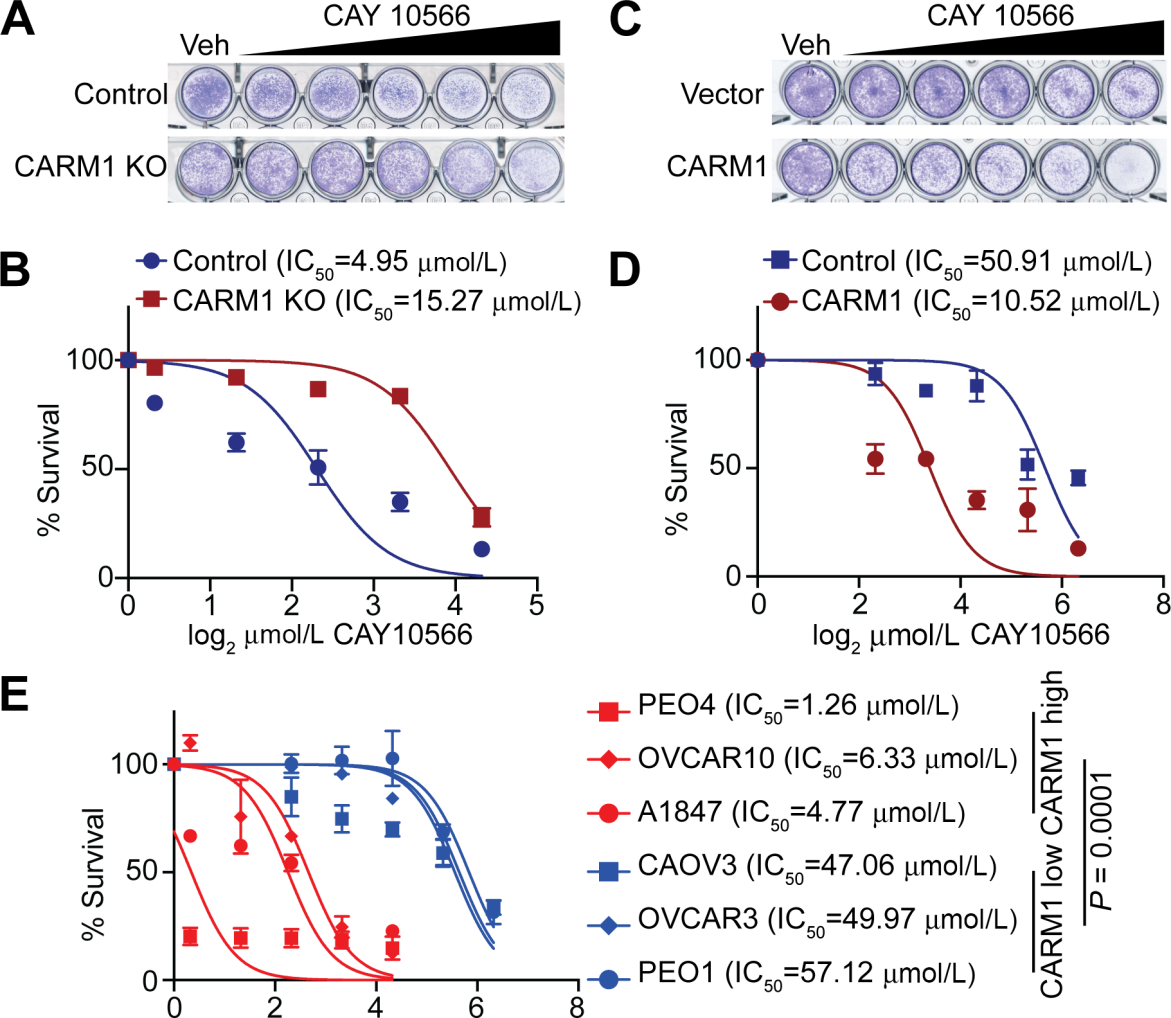
CARM1 expression sensitizes cells to SCD1 inhibition. Sensitivity of control and CARM1 KO A1847 cells to SCD1 inhibitor CAY10566 determined by colony formation assay (**A**), which was quantified as dose response curves (**B**). Sensitivity of control and CARM1-overexpressing CAVO3 cells to SCD1 inhibitor CAY10566 determined by colony formation assay (**C**), which was quantified as dose–response curves (**D**). **E,** Sensitivity of the indicated ovarian cancer cell lines to SCD1 inhibitor CAY10566 determined by colony formation assay, which was quantified as dose–response curves. *P* value was calculated using a two-tailed Student *t* test. Data represent mean ± SEM, *n* = 4 biologically independent experiments.

Because CARM1 promotes MUFAs synthesis through upregulating SCD1, we next investigated whether blocking SCD1 activity is more potent in inhibiting the proliferation of cells expressing CARM1. Accordingly, we treated wildtype and CARM1 knockout A1847 cells with CAY10566, a SCD1 selective inhibitor, that reduces the levels of MUFAs in these cells (Fig. 3C). Indeed, the IC_50_ of CAY10566 is increased by CARM1 knockout in A1847 cells (Fig. 4A and B). Similar observation was also made in PEO4 control and CARM1 knockout cells (Supplementary Fig. S4A and S4B). Contrariwise, CARM1 overexpression sensitized CAOV3 cells to CAY10566 (Fig. 4C and D). Consistently, across a range of ovarian cancer cell lines with established CARM1 expression status (20), IC_50_s of CAY10566 were significantly lower in CARM1-high versus CARM1-low expression cells (Fig. 4E). Thus, we conclude that CARM1 expression status correlates with sensitivity to SCD1 inhibitor CAY10566.

**FIGURE 5:**
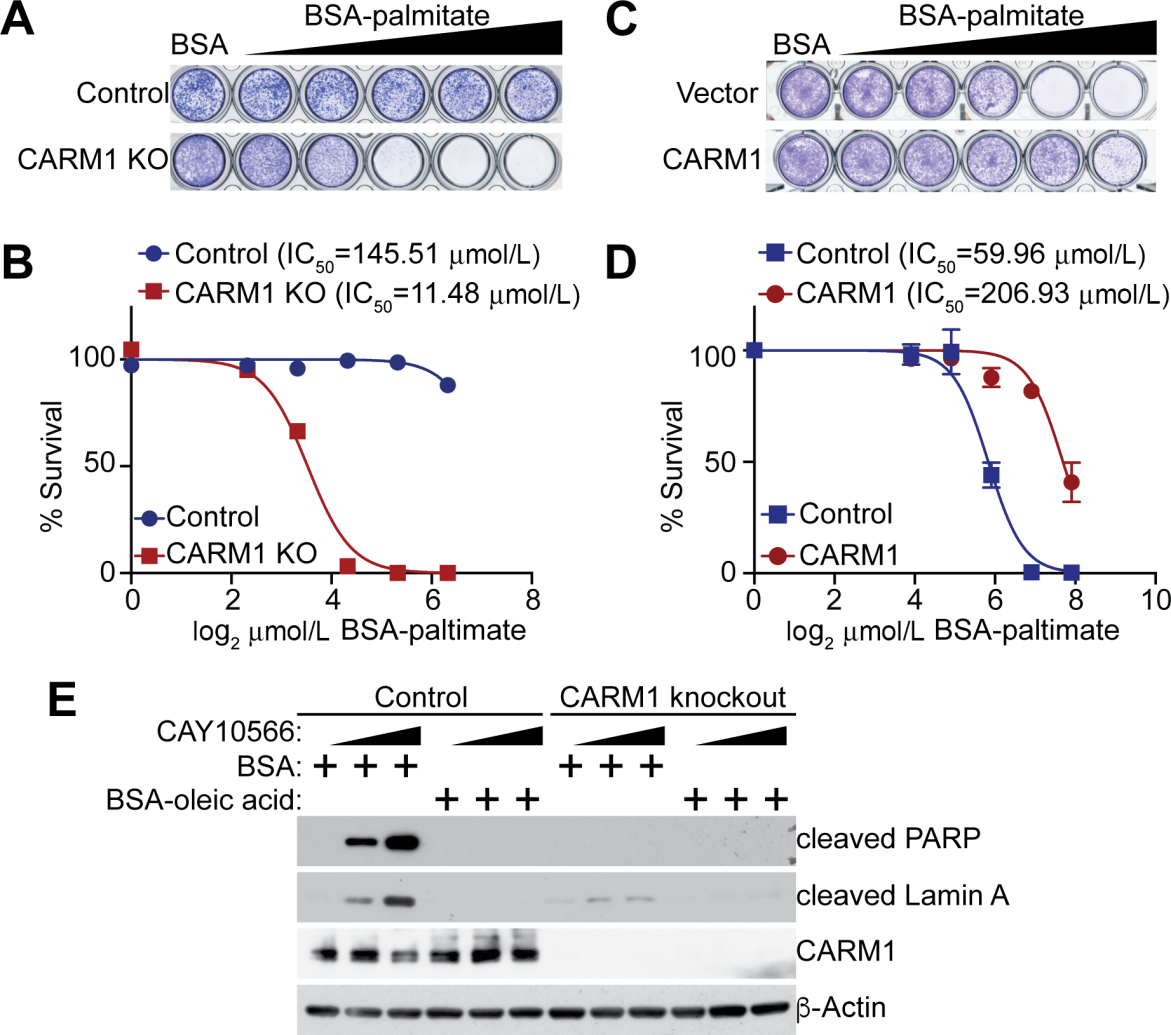
CARM1 KO sensitizes cells to saturated FA. Sensitivity of control and CARM1 KO A1847 cells to BSA conjugated palmitate FA determined by colony formation assay (**A**), which was quantified as dose–response curves (**B**). **C–D**, Same as A and B but not control and CARM1-overexpressing CAVO3 cells. **E,** Expression of cleaved PARP, cleaved Lamin A, CARM1 and a loading control β-actin in control and CARM1 KO A1847 cells treated with or without SCD1 inhibitor CAY10566 and supplemented with 1 mg/mL BSA conjugated oleic acid or BSA control. Data represent mean ± SEM, *n* = 4 biologically independent experiments.

Recent studies suggest that accumulation of saturated FAs contributes to the tumor suppressive effects of SCD1 inhibition (10), which predicts that CARM1 expression will increase the tolerance to saturated FAs. Indeed, CARM1 knockout sensitizes A1847 and PEO4 cells to palmitic saturated FA (Fig. 5A and B; Supplementary Fig. S4C and S4D), which correlates with downregulation of SCD1 by CARM1 knockout (Fig. 1). Conversely, CARM1 overexpression increased the IC_50_ of palmitic saturated FA (Fig. 5C and D). Consistently, SCD1 inhibitor CAY10566 selectively induced expression of apoptotic markers such as cleaved PARP and cleaved Lamin A in control but not CARM1 knockout A1847 cells (Fig. 5E). Notably, addition of oleic acid (18:1) MUFA completely blocked apoptosis induced by CAY10566 (Fig. 5E), indicating the observed effects are due to on target effects of SCD1 inhibition and the associated reduction in MUFAs. Together, we conclude that SCD1 inhibition is selective against CARM1 expression in ovarian cancer cells.

### SCD1 Inhibition Suppresses CARM1-expressing Ovarian Cancer *In Vivo*

**FIGURE 6:**
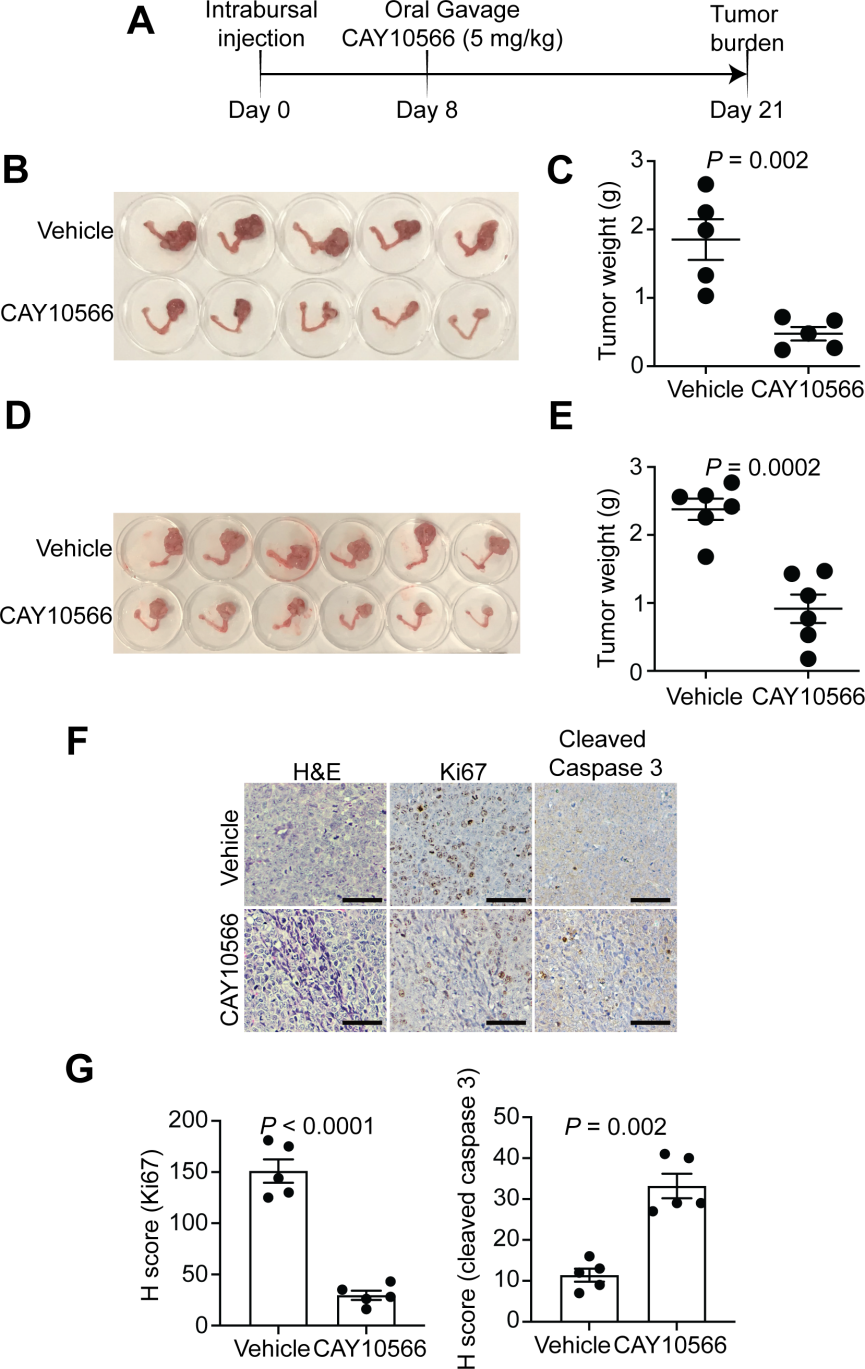
SCD1 inhibition suppresses CARM1-expressing ovarian cancer *in vivo*. **A,** Schematic of experimental design for orthotopic xenograft ovarian cancer mouse model using A1847 cells. **B** and **C,** Reproductive tracts with tumors from the indicated treatment groups were dissected at the end of treatment (*n* = 5 mice per group; B). And the weights of tumors dissected from the indicated groups were measured as a surrogate for tumor burden (C). **D** and **E,** Same as B and C, but for syngeneic ovarian cancer mouse model using CARM1-expressing UPK10 cells (*n* = 5 mice per group). **F** and **G,** Tumors formed by A1847 cells treated with vehicle control or SCD1 inhibitor CAY10566 were subjected to IHC staining for the cell proliferative marker Ki67 and apoptotic marker cleaved caspase 3 (F), which was quantified on the basis of H score (G). *N* = 5 mice per group. Scale bars = 100 μm. Data represent mean ± SEM. *P* values were calculated using two-tailed *t* test.

We next sought to directly test whether SCD1 inhibition suppresses the proliferation of ovarian tumors *in vivo* in a CARM1 status–dependent manner. We employed two distinct mouse models of ovarian cancer. We first used the orthotopic xenograft models produced by CARM1-expressing A1847 cells. The transplanted cells grew for 8 days to establish orthotopic tumors (Fig. 6A). The mice were administrated an oral daily treatment of either vehicle or CAY10566 (5 mg/kg) for 2 weeks (21). By using tumor weight as an indicator of tumor burden, we observed that the burden of orthotopic xenografts formed by CARM1-expressing A1847 cells was significantly reduced by CAY10566 treatment. (Fig. 6B and C). Remarkably, the observed effect in tumor suppression by CAY10566 treatment depends on CARM1 expression. For instance, the growth of tumors formed by CARM1 knockout A1847 cells was not affected by CAY10566 treatment (Supplementary Fig. S5A and S5B). CAY10566 was well tolerated as evidenced by the fact that CAY10566 treatment did not reveal overt abnormalities of liver, pancreas, and kidney based on histologic analyses (Supplementary Fig. S5C).

We also employed a syngeneic mouse model generated by CARM1-expressing UPK10 mouse ovarian cancer cells (5). Consistent with our previous observation in orthotopic xenograft, CAY10566 treatment resulted in a significant reduction in the tumor burden in the syngeneic model (Fig. 6D and E). Consistently, as a control, the burden of tumors formed by CARM1 knockout UPK10 cells was not significantly affected by CAY10566 treatment (Supplementary Fig. S5D–S5G). Together, we conclude that SCD1 inhibition suppresses the growth of ovarian cancers *in vivo* in a CARM1 status–dependent manner.

Next, we determined whether the observed inhibition of tumor growth *in vivo* correlate with the mechanism we identified *in vitro*. Accordingly, we stained for Ki67, a marker for cell proliferation, and cleaved caspase 3, an apoptotic marker, by IHC. Indeed, in CARM1-high tumors, CAY10566 treatment led to a significant decrease of Ki67 and an increase in cleaved caspase 3 (Fig. 6F and G). Validating our *in vitro* findings, expression of ACC1, FASN, and SCD1 was expressed at a significantly lower level in tumors formed by CARM1 knockout A1847 cells compared with controls (Supplementary Fig. S5H and S5I). This was not affected by SCD1 inhibitor CAY10566 treatment (Supplementary Fig. S5H and S5I). Together, these observations support that CAY10566, a SCD1 inhibitor, is effective in suppressing ovarian cancer with high expression if CARM1 *in vivo*, and this effect is correlated with the inhibition of cell proliferation and the induction of apoptosis.

## Discussion

As a transcriptional coactivator, CARM1 is known to regulate gene expression through its enzymatic activity (4). We show that CARM1 regulates FA metabolism independently of its enzymatic activity through enhancing the recruitment of ER stress response transcription factor XBP1s. Thus, CARM1 contributes to cancer by both enzymatic activity–dependent and enzymatic activity–independent functions. A limitation of our study lies in the fact that CARM1 may regulate additional pathways beyond FA metabolism. Regardless, our results clearly demonstrated that FA metabolism represents a vulnerability of CARM1-expressing ovarian cancer, which can be therapeutically explored by targeting SCD1.

Consistent with our finding, a previous study reported a role of CARM1 in promoting FA *de novo* synthesis in neurodegenerative diseases by upregulating *ACC* mRNA at the transcription level (22). Thus, it is likely that CARM1 may reprogram FA metabolism in various pathologic conditions. Another limitation of our study is that we did not perform a targeted metabolomics analysis of the tumors treated by the SCD1 inhibitor. Thus, future studies are warranted to examine how SCD1 inhibition affects FA metabolism in a CARM1-dependent manner *in vivo* in mouse models.

We showed that SCD1 inhibition suppresses the growth of CARM1-expressing ovarian tumor cells both *in vitro* and *in vivo*. CARM1 upregulates the FA metabolism and SCD1 inhibition causes accumulation of saturated FAs in CARM1-expressing cells. Thus, our findings are consistent with recent studies that accumulation of saturated FAs is tumor suppressive effects of SCD1 inhibition (10). In addition, there is evidence to support that tumor suppressive effects of SCD1 are exacerbated by low lipid or high saturated FAs (10). This raises the possibility that diets or interventions that lowers lipid to increases saturated FAs may further improve the antitumor effects of SCD1 inhibition–based therapeutic approaches for several cancer types with frequent CARM1 overexpression and amplification including ovarian cancer (6–8).

## Supplementary Material

Figure S1 CARM1 regulates genes involved in fatty acid synthesis pathway.

Figure S2 CARM1 regulates FASN and ACC1 expression by recruiting XBP1s to their promoters.

Figure S3 CARM1 reprograms lipid metabolism.

Figure S4 CARM1 expression confers the sensitivity to SCD1 inhibition.

Figure S5 SCD1 inhibition suppresses CARM1-expressing ovarian cancer in vivo.

Table S1 Reverse Phase Protein Assay (RPPA) analysis in control and CARM1 knockout A1847 cells.

Table S2 Reverse Phase Protein Assay (RPPA) analysis in control and CARM1 knockout PEO4 cells.
