# Supplementary material for: Targeting Fatty Acid Reprogramming Suppresses CARM1-expressing Ovarian Cancer
Source: Cancer Res Commun. 2023 Jun 20;3(6):1067–77. doi: 10.1158/2767-9764.CRC-23-0030 (PMC10281290; doi:10.1158/2767-9764.CRC-23-0030)
Supplement: Figure S1 — CARM1 regulates genes involved in fatty acid synthesis pathway. [file crc-23-0030-s01.pdf]

Lombardi et al Figure S1

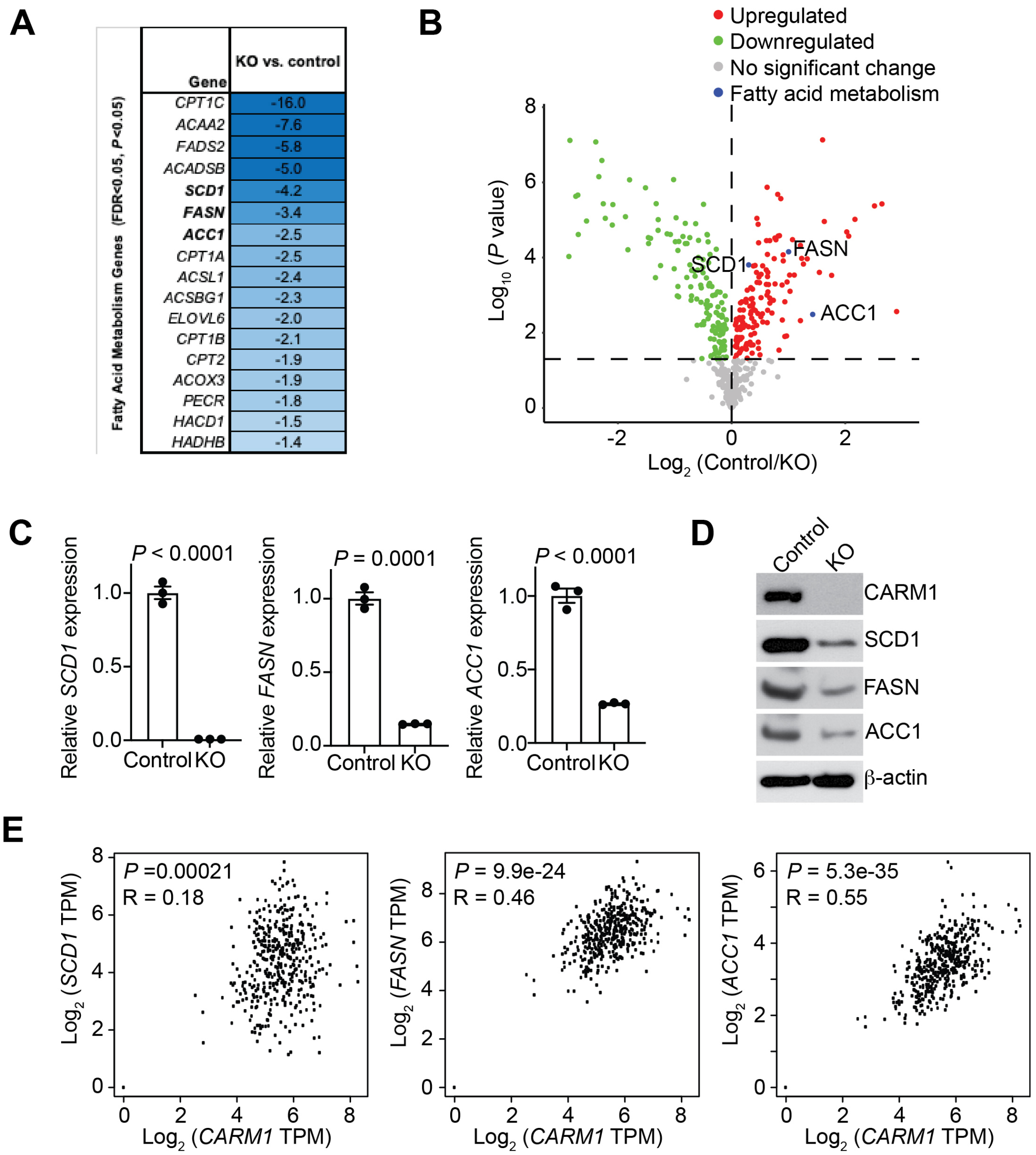

Supplementary Figure 1. CARM1 regulates genes involved in fatty acid synthesis pathway.
A, Heatmap of the fold-changes of expressed genes in fatty acid metabolism pathway that are downregulated by CARM1 knockout in A1847 cells.
B, Volcano plot illustrating the protein expression detected by Reverse Phase Protein Assay (RPPA) in wildtype or CARM1 knockout PEO4 cell lysates. Red dots and green dots indicate protein expression significantly upregulated in wildtype and knockout cells, respectively. SCD1, FASN and ACC1 were highlighted in blue. *P* value was calculated using a two-tailed Student *t* test.
C, RT-qPCR analysis of SCD1, FASN and ACC1 expression in control and CARM1 knockout PEO4 cells. *P* values were calculated using a two-tailed t test.
D, Expression of CARM1, SCD1, FASN and ACC1 in control and CARM1 knockout PEO4 cells was determined by immunoblot. β-actin was used as a loading control.
E, Correlation between CARM1 and SCD1, FASN or ACC1 in the high-grade serous ovarian cancer TCGA dataset. *P* values were determined by Pearson correlation analysis.
Data represent mean ± SEM, n = 3 biologically independent experiments.
