# Supplementary material for: Targeting Fatty Acid Reprogramming Suppresses CARM1-expressing Ovarian Cancer
Source: Cancer Res Commun. 2023 Jun 20;3(6):1067–77. doi: 10.1158/2767-9764.CRC-23-0030 (PMC10281290; doi:10.1158/2767-9764.CRC-23-0030)
Supplement: Figure S2 — CARM1 regulates FASN and ACC1 expression by recruiting XBP1s to their promoters. [file crc-23-0030-s02.pdf]

Lombardi et al Figure S2

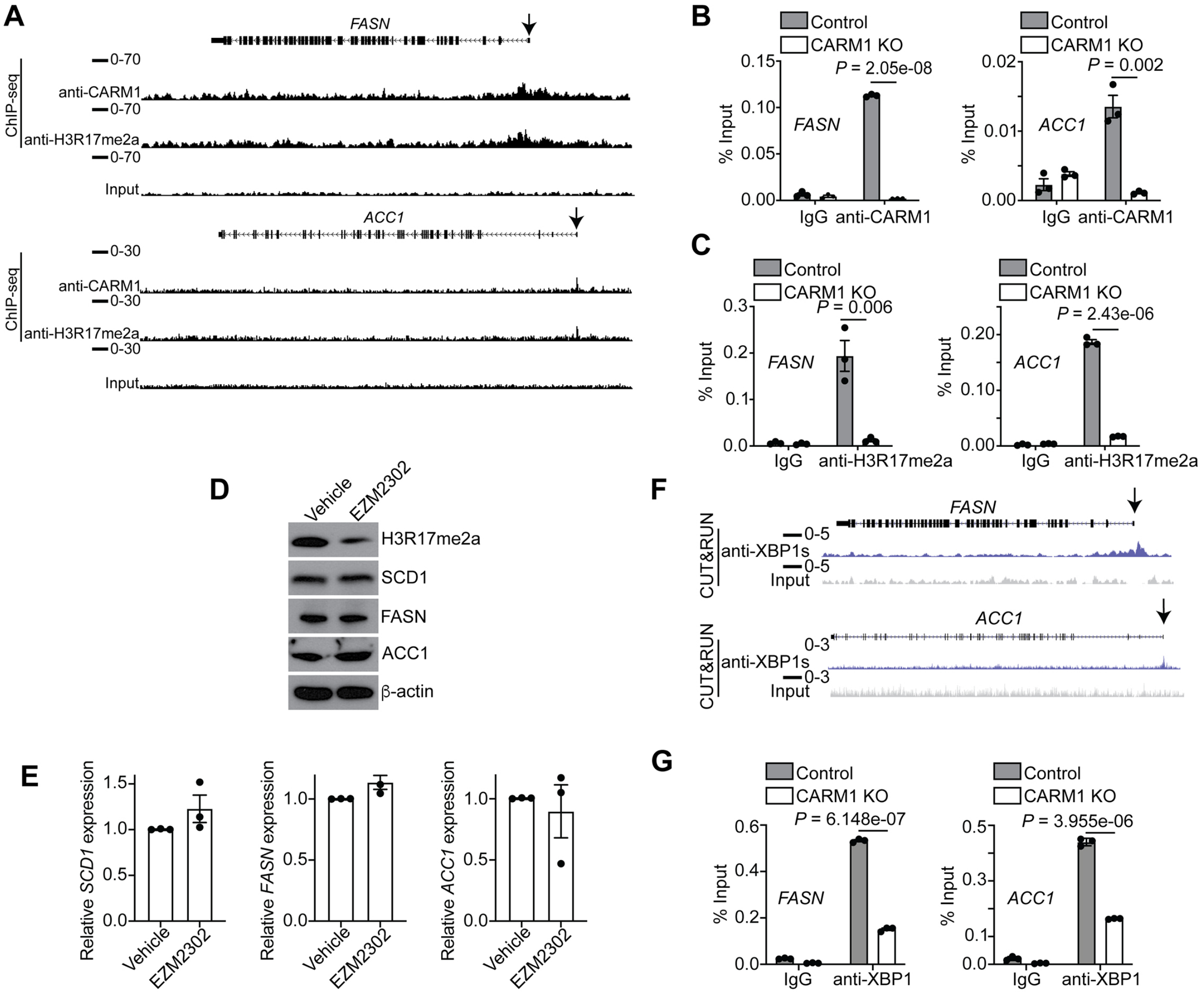


**Supplementary Figure 2.** CARM1 regulates *FASN* and *ACC1* expression by recruiting XBP1s to their promoters.

**A**, ChIP-seq tracks of CARM1 and H3R17me2a in the *FASN* and *ACC1* promoters. The arrows indicate the CARM1 and H3R17me2a peak in the promoter region of *FASN* and *ACC1* genes.

**B-C,** ChIP-qPCR analysis of the binding of CARM1 (**B**) and H3R17me2a (**C**) at the *FASN* and *ACC1* gene promoters. An isotype matched IgG was used as a negative control.

**D,** Expression of H3R17me2, SCD1, FASN and ACC1 in A1847 cells treated with vehicle or CARM1 methyltransferase inhibitor EZM2302 was determined by immunoblot. β-actin was used as a loading control.

**E**, Same as D, but analysis for expression of *SCD1*, *FASN* and *ACC1* by RT-qPCR analysis.

**F**, CUT&RUN tracks of XBP1s in the *FASN* and *ACC1* promoters. The arrows indicate the XBP1s peaks in the promoter region of *FASN* and *ACC1* genes.

**F**, ChIP-qPCR analysis for the binding of XBP1s or negative control IgG at the *FASN* and *ACC1* gene promoter.

*P* value was calculated using a two-tailed Student *t* test. Data represent mean ± SEM, n = 3 biologically independent experiments.
