# Supplementary material for: Targeting Fatty Acid Reprogramming Suppresses CARM1-expressing Ovarian Cancer
Source: Cancer Res Commun. 2023 Jun 20;3(6):1067–77. doi: 10.1158/2767-9764.CRC-23-0030 (PMC10281290; doi:10.1158/2767-9764.CRC-23-0030)
Supplement: Figure S3 — CARM1 reprograms lipid metabolism. [file crc-23-0030-s03.pdf]

Lombardi et al Figure S3

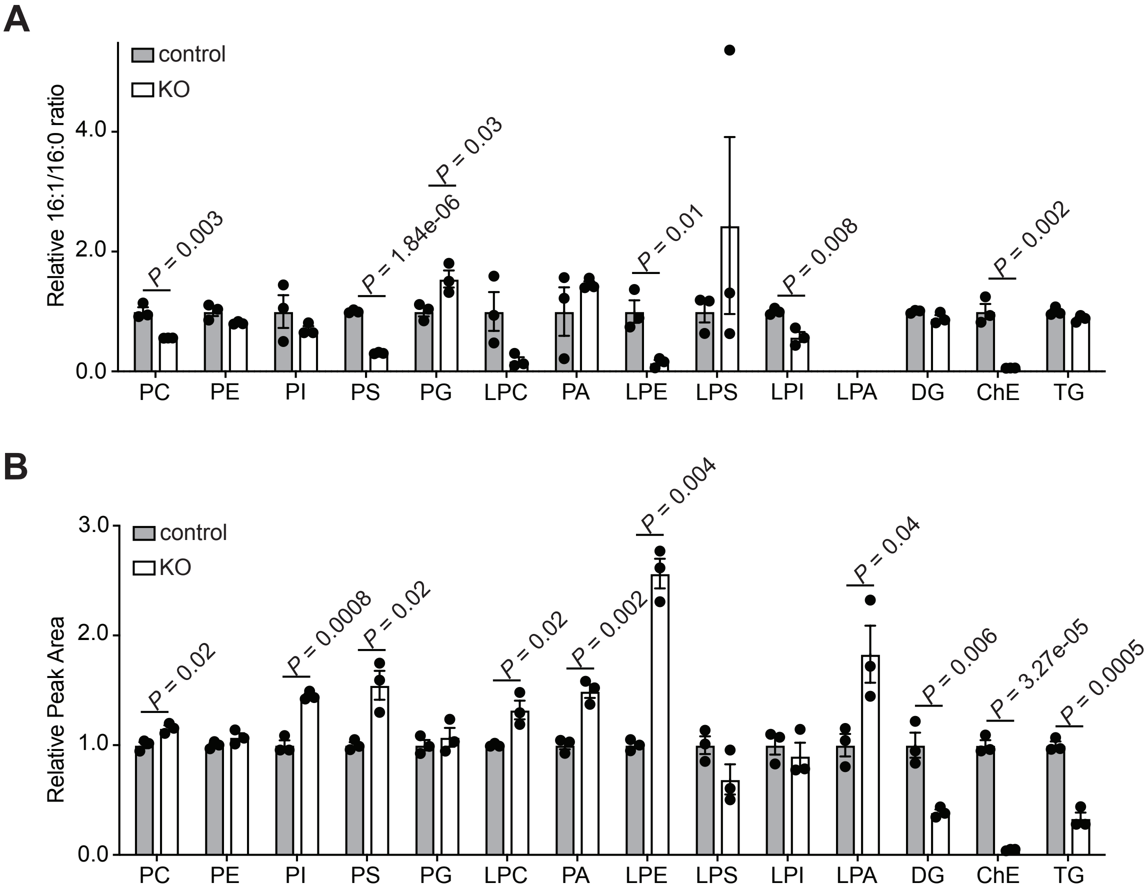

Supplementary Figure 3. CARM1 reprograms lipid metabolism.

A, The ratio between palmitic acid (16:0) and palmitoleic acid (16:1) in different lipid species determined by lipid profiling in control and CARM1 knockout A1847 cells.

B, The levels of different lipid species determined by lipid profiling in control or CARM1 knockout A1847 cells.

PC: Phosphatidylcholine; PE: phosphatidylethanolamine; PI: Phosphatidylinositol; PS: Phosphatidylserine; PG: Phosphatidylglycerol; LPC: Lysophosphatidylcholine; PA: Phosphatidic acid; LPE: lysophosphatidylethanolamine; LPS: Lysophosphastidylserine; LPI: Lysophosphatidylinositol; LPA: Lysophosphatidic acid; DG: Diglyceride; ChE: cholesterol ester; TG: Triglyceride.

P value was calculated using a two-tailed Student *t* test. Data represent mean ± SEM, n = 3 biologically independent experiments.
