# Supplementary material for: Targeting Fatty Acid Reprogramming Suppresses CARM1-expressing Ovarian Cancer
Source: Cancer Res Commun. 2023 Jun 20;3(6):1067–77. doi: 10.1158/2767-9764.CRC-23-0030 (PMC10281290; doi:10.1158/2767-9764.CRC-23-0030)
Supplement: Figure S4 — CARM1 expression confers the sensitivity to SCD1 inhibition. [file crc-23-0030-s04.pdf]

Lombardi et al Figure S4

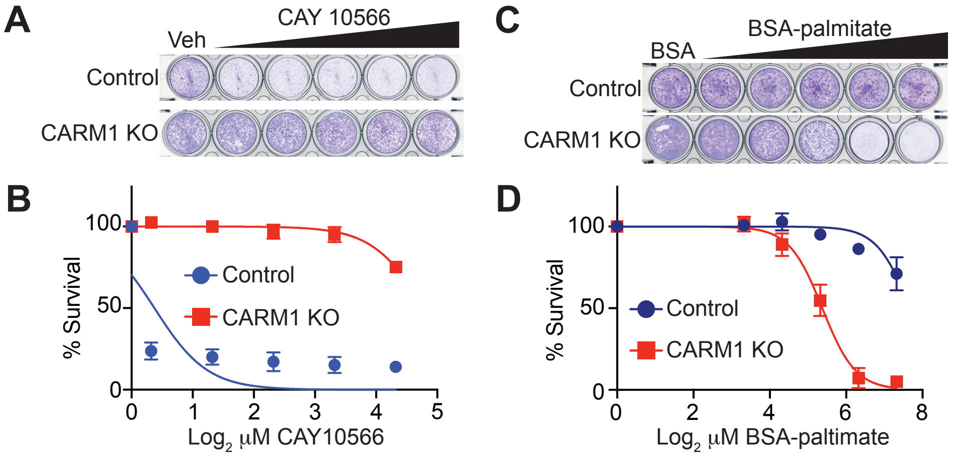


**Supplementary Figure 4.** CARM1 expression confers the sensitivity to SCD1 inhibition. **A-B,** Sensitivity of control and CARM1 knockout PEO4 cells to SCD1 inhibitor CAY10566 determined by colony formation assay (**A**), which was quantified as dose response curves (**B**). **C-D,** Sensitivity of control and CARM1 knockout PEO4 cells to BSA conjugated palmitate fatty acid determined by colony formation assay (**C**), which was quantified as dose response curves (**D**). *P* value was calculated using a two-tailed Student *t* test. Data represent mean ± SEM, n = 4 biologically independent experiments.
