# Supplementary material for: Targeting Fatty Acid Reprogramming Suppresses CARM1-expressing Ovarian Cancer
Source: Cancer Res Commun. 2023 Jun 20;3(6):1067–77. doi: 10.1158/2767-9764.CRC-23-0030 (PMC10281290; doi:10.1158/2767-9764.CRC-23-0030)
Supplement: Figure S5 — SCD1 inhibition suppresses CARM1-expressing ovarian cancer in vivo. [file crc-23-0030-s05.pdf]

Lombardi et al Figure S5

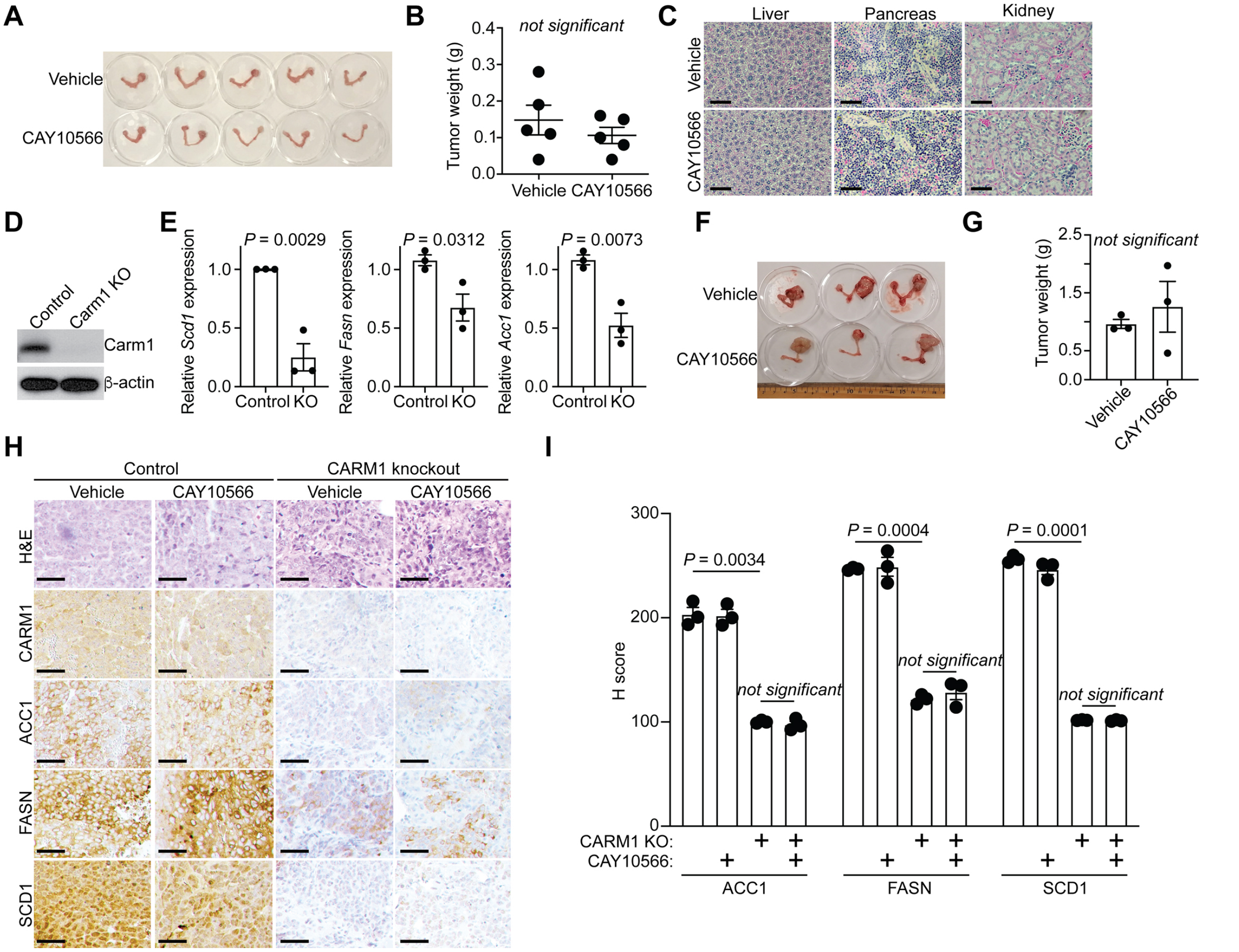

Supplementary Figure 5. SCD1 inhibition suppresses CARM1-expressing ovarian cancer *in vivo*.

A-B, Mice bearing orthotopic CARM1 knockout tumors were randomized into two indicated treatment groups. Reproductive tracts with tumors from the indicated treatment groups were dissected at the end of treatment (n=5 mice per group) (**A**). The weights of tumors dissected from the indicated groups were measured as a surrogate for tumor burden (**B**).

C, Representative H&E staining of liver, pancreas and kidney sections from tumor treated with vehicle and SCD1 inhibitor CAY10566. Bars = 100 μm.

D-E, Expression of CARM1 and a loading control β-actin in control and *Carm1* knockout UPK10 cells was determined by immunoblot (**D**). And expression of *SCD1*, *FASN* and *ACC1* in control and Carm1 knockout UPK10 cells was determined by RT-qPCR analysis (**E**).

F-G, Reproductive tracts with tumors formed by Carm1 knockout UPK10 cells from the indicated treatment groups were dissected at the end of treatment (n=3 mice per group) (**F**). And the weights of tumors dissected from the indicated groups were measured as a surrogate for tumor burden (**G**).

H-I, Tumors formed by A1847 control or CARM knockout cells treated with vehicle control or SCD1 inhibitor CAY10566 were subjected to immunohistochemical staining for the indicated proteins (**H**), which was quantified based on histological score (H score) (**I**). N=5 mice per group. Scale bars = 100 μm.

Data represent mean ± SEM. *P* values were calculated using a two-tailed t test.
